# Kallikrein-related peptidase-4 (KLK4): role in enamel formation and revelations from ablated mice

**DOI:** 10.3389/fphys.2014.00240

**Published:** 2014-07-04

**Authors:** John D. Bartlett, James P. Simmer

**Affiliations:** ^1^Harvard School of Dental MedicineBoston, MA; ^2^Department of Mineralized Tissue Biology, The Forsyth InstituteCambridge, MA; ^3^Department of Biological and Material Sciences, University of Michigan School of DentistryAnn Arbor, MI, USA

**Keywords:** enamel development, MMP20, amelogenin, ameloblastin, enamelin, enamel crystallites, enamel rods

## Abstract

Enamel development occurs in stages. During the secretory stage, a soft protein rich enamel layer is produced that expands to reach its final thickness. During the maturation stage, proteins are removed and the enamel matures into the hardest substance in the body. KLK4 is expressed during the transition from secretory to the maturation stage and its expression continues throughout maturation. KLK4 is a glycosylated chymotrypsin-like serine protease that cleaves enamel matrix proteins prior to their export out of the hardening enamel layer. Mutations in *KLK4* can cause autosomal recessive, non-syndromic enamel malformations in humans and mice. *Klk4* ablated mice initially have normal-looking teeth with enamel of full thickness. However, the enamel is soft and protein-rich. Three findings are notable from *Klk4* ablated mice: first, enamel rods fall from the interrod enamel leaving behind empty holes where the enamel fractures near the underlying dentin surface. Second, the ~10,000 crystallites that normally fuse to form a solid enamel rod fail to grow together in the ablated mice and can fall out of the rods. Third, and most striking, the crystallites grow substantially in width and thickness (a- and b-axis) in the ablated mice until they almost interlock. The crystallites grow in defined enamel rods, but interlocking is prevented presumably because too much protein remains. Conventional thought holds that enamel proteins bind specifically to the sides of enamel crystals to inhibit growth in width and thickness so that the thin, ribbon-like enamel crystallites grow predominantly in length. Results from *Klk4* ablated mice demonstrate that this convention requires updating. An alternative mechanism is proposed whereby enamel proteins serve to form a mold or support structure that shapes and orients the mineral ribbons as they grow in length. The remnants of this support structure must be removed by KLK4 so that the crystallites can interlock to form fully hardened enamel.

## Introduction

The enamel layer covers the crown of the tooth and is unique because it is an epithelially-derived calcified tissue and becomes the hardest substance in the body. Its hardness is between that of iron and carbon steel, but enamel has a higher elasticity (Newbrun and Pigman, 1960). Enamel hardness is a function of its high mineral content. Unlike bone and dentin (20–30% organic material by weight), fully formed enamel contains very little protein (less than 1% organic material) (Lefevre and Manly, 1938; Deakins and Volker, 1941). Enamel mineral is very similar to hydroxyapatite (HAP) [Ca_5_OH (PO_4_)_3_], but also contains low percentages of carbonate, sodium, and magnesium. Therefore within the body, teeth are the most resistant to deterioration and have been examined extensively for anthropological studies.

But, what are the developmental and mechanistic processes that make enamel harder than the cementum formed along the tooth root, the dentin underlying the enamel layer, and the skeletal bones? The ameloblasts are a single cell layer that cover the developing enamel and are responsible for enamel composition. Enamel development (amelogenesis) can be broken down into three defined stages: secretory, transition and maturation. The stages are defined by the morphology and function of the ameloblasts. Dentin mineralizes first and pre-ameloblasts transform into secretory stage ameloblasts by elongating into tall columnar cells and by forming Tomes' processes at their apical ends nearest the forming enamel. The Tomes' process is a conical structure that points toward the forming enamel matrix. Enamel matrix proteins are primarily secreted from one side of the Tomes' process (secretory face) and all ameloblasts within a row secrete protein from the same side of their Tomes' processes. The first formed enamel ribbons grow between the dentin crystals perhaps by mineralizing around dentin proteins such as collagen. At their growing tips near the Tomes' process, secretory stage enamel ribbons are only about 1.5 nm thick and 15 nm wide (Daculsi and Kerebel, 1978; Cuisinier et al., 1992) and these ribbons are extended until they span the entire thickness of the enamel layer. Approximately 10,000 parallel mineral ribbons are present in each enamel rod (Daculsi et al., 1984). A rod is about 5 μm in cross-sectional diameter (Skobe and Stern, 1980) and each is generated by a single ameloblast (Skobe, 1977). Enamel ribbons elongate at the mineralization front where enamel proteins are secreted (Ronnholm, 1962). As the ameloblasts secrete large amounts of enamel matrix proteins, they move away from the dentin surface so that the nascent enamel layer can thicken. The mineral ribbons crystallize into HAP within the rod and will grow progressively in c-axis length parallel to one another as the ameloblasts move progressively away from the dentin surface. The crystallites are surrounded with abundant proteins that prevent them from fusing into a solid rod. The secretory stage enamel is therefore protein rich and has a soft cheese-like consistency.

During the secretory stage, ameloblasts not only move away from the dentin as the enamel thickens, but they also move in groups that slide by one another and this movement culminates in the characteristic decussating enamel prism pattern observed in rodent incisors (Reith and Ross, 1973) or the entwined gnarled prism pattern seen in human molars (Boyde, 1989). Secretory stage ameloblasts secrete four different proteins into the enamel matrix. Three are “structural” proteins and one is a proteinase. The structural proteins are amelogenin (AMELX), ameloblastin (AMBN), enamelin (ENAM), and the proteinase is matrix metalloproteinase-20 (MMP20, enamelysin). Amelogenin comprises approximately 80–90% of the organic matter within the secretory stage enamel matrix and ameloblastin and enamelin comprise roughly 5 and 3–5%, respectively (Fincham et al., 1999; Hu et al., 2007). MMP20 is present in trace amounts. The precise function of these proteins remains unclear. However, human mutations in *AMELX* (Hu et al., 2012), *ENAM* (Rajpar et al., 2001), and *MMP20* (Kim et al., 2005; Ozdemir et al., 2005; Lee et al., 2010; Gasse et al., 2013) genes and mouse knockout models (Gibson et al., 2001; Caterina et al., 2002; Fukumoto et al., 2004; Masuya et al., 2005; Seedorf et al., 2007; Hu et al., 2008) have definitively demonstrated that each of these proteins are absolutely required for proper enamel formation. This conclusion is supported by the observation that the genes encoding secretory stage enamel proteins are consistently pseudogenized in vertebrates that have lost the ability to make teeth, or specifically dental enamel, during evolution (Meredith et al., 2009, 2011, 2013). By the end of the secretory stage the enamel layer has achieved its full thickness. It is not until the end of the maturation stage when the proteins are almost completely removed, that the enamel achieves its final hardened form.

As the ameloblasts enter the transition stage, they no longer move relative to each other. They retract their Tomes' processes and transition into shorter and fatter maturation stage cells and, at the enamel surface, start modulating between ruffle and smooth-ended cells (Smith, 1998). It is during the maturation stage that ameloblasts actively secrete kallikrein-related peptidase-4 (KLK4) to help remove the mass of previously secreted and partially hydrolyzed (by MMP20) matrix proteins from the enamel layer so that the crystallites can expand, coalesce and fuse with adjacent crystals (Simmer et al., 2009). This strengthens the enamel rods and forms the most highly mineralized substance in the body. Therefore, unlike cementum, dentin and bone, the proteins responsible for mineralization are removed from enamel so that the enamel can attain its final hardened form. Also, relative to the other mineralized tissues, the entwined rod and interrod enamel forms a more highly ordered 3D structure that is highly resistant to occlusal forces. The stages of enamel development and the developmental processes as the enamel forms are remarkably similar in different mammalian species (Weinmann et al., 1942; Robinson et al., 1988). Rodents have continuously erupting incisors so every developmental stage is always present in specific locations along the forming rodent incisor.

## Enamel proteinases

The proteinase expressed during the secretory through early maturation stage is MMP20 (Begue-Kirn et al., 1998) and the proteinase expressed from the transition through maturation stages is KLK4 (Hu et al., 2002). To date, these are the only two proteinases proven to be secreted into the enamel matrix (Bartlett, 2013). Both proteinases are present in small amounts during enamel development and each proteinase was separately cloned by performing PCR-based homology cloning (Bartlett et al., 1996; Simmer et al., 1998). KLK4 was originally named enamel matrix serine proteinase-1 (EMSP1) (Simmer et al., 1998), but its name was changed to KLK4 because the gene encoding KLK4 locates within the kallikrein gene cluster.

Human and mouse mutations in both *MMP20* (Caterina et al., 2002; Kim et al., 2005; Ozdemir et al., 2005; Lee et al., 2010; Gasse et al., 2013) and *KLK4* (Hart et al., 2004; Simmer et al., 2009; Wang et al., 2013) cause severe enamel malformations and therefore demonstrate that no other proteinase has an extensive overlapping function with either of these proteinases. If this were the case, no severe enamel phenotype would likely occur if the activity of MMP20 or KLK4 were compromised. Furthermore, accumulated porcine secretory stage enamel protein cleavage products have been extensively characterized and MMP20 specifically cleaves recombinant enamel proteins *in vitro* to generate the full spectrum of cleavages that occur *in vivo* (Ryu et al., 1999; Nagano et al., 2009). Others have proposed that MMP9 is present within secretory stage developing enamel (Feng et al., 2012). However, this claim is uncertain as MMP20 activity can explain all of the secretory stage amelogenin cleavages and MMP9 mutations cause metaphyseal anadysplasia, which is not associated with enamel defects (Lausch et al., 2009). Similarly, chymotrypsin C is associated with enamel formation (Lacruz et al., 2011). However, although loss of *CTRC* function is a risk factor for pancreatitis, an associated enamel phenotype has not been described (Zhou and Sahin-Toth, 2011). Signal-peptide-peptidase-like 2a (SPPL2A) is a membrane bound protease in lysosomes/late endosomes that is expressed by enamel epithelium during the secretory and maturation stages of amelogenesis. Spll2a null mice show defective enamel, highlighting the importance of intracellular degradation of enamel proteins reabsorbed by endocytosis (Bronckers et al., 2013). Although it is likely that several proteases degrade enamel proteins within ameloblast lysosomes, MMP20, and KLK4 remain the only proteinases that are known to be secreted into the enamel matrix (Bartlett, 2013).

## Discovery of KLK4

In 1977 a protease was purified from pig enamel (Fukae et al., 1977) that was later demonstrated to be inhibited by serine proteinase inhibitors phenylmethylsulfonyl fluoride (PMSF) and diisopropylfluoro phosphate (DIFP) (Shimizu et al., 1979). This protease was expressed during the early maturation stage when the enamel proteins are reabsorbed from the hardening enamel (Overall and Limeback, 1988). KLK4 was eventually cloned by PCR-based homology cloning from porcine cDNA with subsequent screening of a porcine cDNA library (Simmer et al., 1998). The porcine KLK4 preproenzyme is composed of 254 amino acids while the proenzyme has 230 residues and the active form has 224 amino acids (Simmer et al., 1998). The *KLK4* genes of both mouse and human have six exons the first of which is non-coding. The mouse *Klk4* gene is approximately 10 kb in size and locates in cytogenic region B2 on mouse chromosome 7 (Hu et al., 2000b). The human *KLK4* gene is approximately 7 kb in size and is located near the telomere of chromosome 19 (19q13.3–19q13.4) in a cluster of genes including the KLK family of serine proteases. Its gene exon/intron structure and protein domain structure is identical to that of the mouse (Hu et al., 2000b). Thus, because KLK4/EMSP1 was cloned after MMP20, it became the second proteinase identified by name that is secreted into the developing enamel matrix.

## KLK4 tissue localization

**Figure 1:**
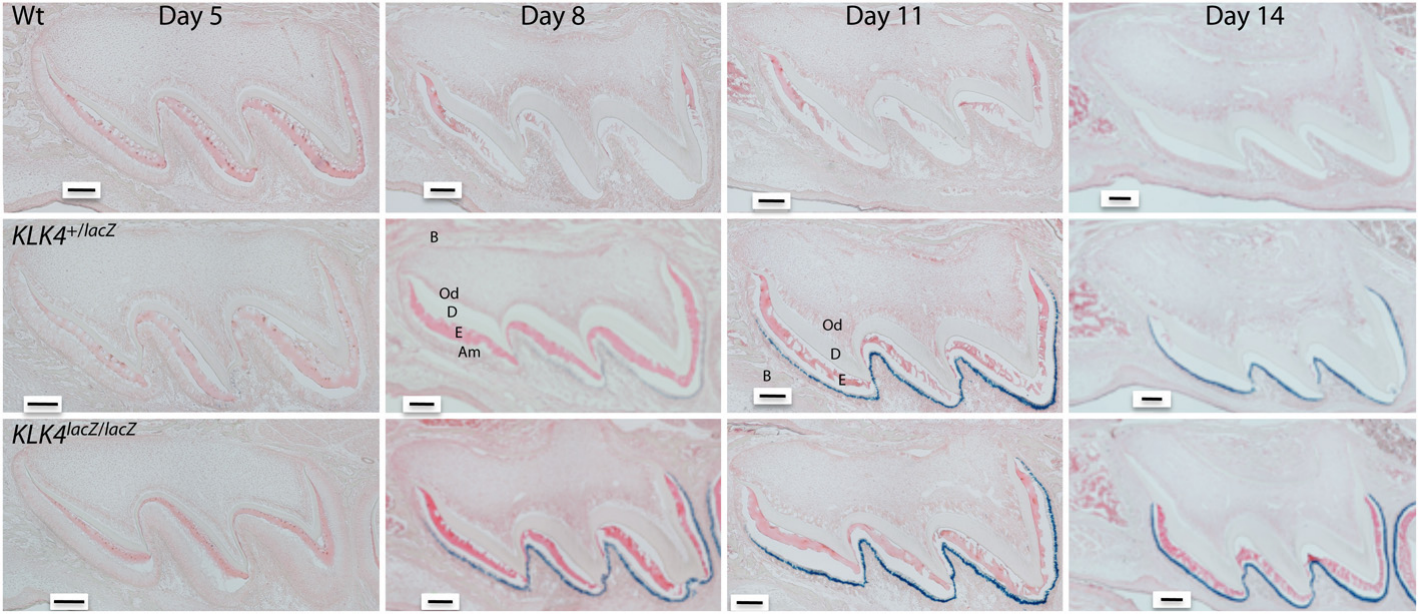
**Histochemical detection of β-galactosidase activity in maxillary first molars of wild-type (Wt) heterozygous (*Klk4^+^*^/lacZ^) and homozygous (*K*lk4^lacZ/lacZ^) mice at postnatal days 5, 8, 11, and 14**. LacZ histochemistry shows nuclear localized β-galactosidase activity where KLK4 is normally expressed. At the 5-h incubation used, no endogenous (lysosomal) β-gal activity was observed and the Wt mice were negative. In mouse molars, a positive signal was only observed in transition and maturation ameloblasts. No expression was observed in odontoblasts. B, Bone; Od, odontoblasts; D, dentin; E, enamel; Am, ameloblasts. Scale bars = 100 μm. This figure was reprinted with permission (S. Karger AG, Basel) from Simmer et al. (2011a).

KLK4 is a glycosylated, chymotrypsin-like serine protease that is expressed and secreted by transition to maturation stage ameloblasts (Hu et al., 2000a,b, 2002). KLK4 protein has not been isolated from any tissue other than from developing teeth (Ryu et al., 2002; Nagano et al., 2009). However, several studies have performed immunoassays or qPCR techniques to identify KLK4 in various tissues and many of these studies conflict with one another as to exactly where KLK4 is expressed (reviewed in Simmer et al., 2011b). To definitively identify where KLK4 is expressed, a gene targeted mouse strain was developed. These mice have a *LacZ* reporter gene with a mouse nuclear localization signal (NLS-βgal) inserted at the natural *Klk4* translation initiation site. Therefore, with these mice, locations of KLK4 expression were identified within tissues by using β-galactosidase histochemistry (Simmer et al., 2009). KLK4 was expressed highly in maturation stage ameloblasts (Figure 1) and low levels of KLK4 expression were observed in the striated ducts of the submandibular salivary gland and in small patches of prostate epithelia. Furthermore, in these *Klk4 LacZ* knock-in mice, no obvious morphological abnormalities were observed in any of the non-dental tissues examined suggesting that their normal development is not *Klk4* dependent (Simmer et al., 2011b). As is true for MMP20, it appears that the only essential, non-overlapping function of KLK4 is in enamel development.

## KLK4 activation

It is not known how KLK4 is activated *in vivo*. Removal of the KLK4 propeptide is essential for activation because it allows a salt linkage to form between the new N-terminus and the side chain of Asp194 and this is essential for enzyme activity (Scully et al., 1998; Debela et al., 2006). Unlike the other kallikrein-related peptidases, KLK4 has a Gln as the last residue of its propeptide and not an Arg or Lys which means that KLK4 cannot be activated by trypsin-like enzymes (Lundwall and Brattsand, 2008). KLK4 cannot activate itself, but can be activated by MMP20 and thermolysin *in vitro* (Ryu et al., 2002). However, KLK4 is active in *Mmp20* ablated mice (Yamakoshi et al., 2011) so MMP20 cannot be the sole KLK4 activator. Previously it was shown that dipeptidyl peptidase I (Cathepsin C, CTSC) activates KLK4 *in vitro* (Tye et al., 2009). In the enamel organ, CTSC is expressed at progressively increasing levels as development progresses to the early maturation stage when KLK4 begins its expression. Therefore, it remains a possibility that this cysteine aminopeptidase is the primary enzyme that activates KLK4.

## KLK4 substrate specificity

KLK4 was assessed for its substrate specificity by using recombinant KLK4 to screen tetrapeptide positional scanning synthetic combinatorial libraries (PS-SCL) (Matsumura et al., 2005). The identified preferred P1–P4 positions were: P1-Arg; P2-Gln/Leu/Val; P3-Gln/Ser/Val and P4-Ile/Val. The first report demonstrating that KLK4 cleaves amelogenin, used native porcine KLK4 incubated with recombinant pig amelogenin and this resulted in the generation of twelve cleavage products which were characterized by N-terminal sequencing (Ryu et al., 2002). It was subsequently demonstrated that the primary MMP20 N-terminal cleavage product, tyrosine-rich amelogenin polypeptide (TRAP), was further cleaved by KLK4 which was consistent with the notion that KLK4 cleaves enamel matrix proteins into small peptides to facilitate their export out of the enamel as the enamel hardens (Nagano et al., 2009). Porcine ameloblastin was stably expressed and secreted from HEK293-N cells and was purified for digestion by KLK4. The cleavage products were characterized by N-terminal sequencing and KLK4 was shown to cleave ameloblastin at nine different sites (Chun et al., 2010). The 32 kDa enamelin is presumed to be an MMP20 cleavage product and it is the only domain of the parent protein that accumulates in the deeper, more mature enamel layer. Native porcine KLK4 was incubated with native porcine 32 kDa enamelin and the digestion products were fractionated by reverse-phase high-performance liquid chromatography (RP-HPLC) and characterized by Edman sequencing, amino acid analysis, and mass spectrometry. KLK4 digestion of the 32-kDa enamelin generated nine major cleavage products (Yamakoshi et al., 2006). Therefore, KLK4 cleaves all the structural enamel matrix proteins that are known to be secreted into the enamel matrix. Recently, it was confirmed that MMP20 activates pro-KLK4 and strikingly, that active KLK4 cleaves and inactivates MMP20 (Yamakoshi et al., 2013). In effect, by activating KLK4, MMP20 inactivates itself. This mechanism of MMP20 inactivation is supported by evidence demonstrating that in *Klk4* ablated mice, MMP20 is active well into the maturation stage when MMP20 activity has normally ceased (Yamakoshi et al., 2011).

## Human *KLK4* mutations

Two different human *KLK4* mutations are known to cause autosomal recessive hypomaturation *amelogenesis imperfecta*. The first discovered is a nonsense mutation occurring upstream of the KLK4 catalytic domain (p.Trp153^*^). This tryptophan residue is completely conserved in mouse and pig KLK4 and expression of this mutated gene would result in a truncated protein lacking the final 101 amino acids which includes the catalytic triad (His71, Asp116, and Ser207). This homozygous mutation occurred in two female siblings and both their primary and permanent dentitions were similarly affected. The sibling's teeth were yellow-brown in color and were excessively sensitive to hot and cold. The enamel was normal in thickness, but radiographically showed only a slight increase in opacity over that of the underling dentin indicating a decreased enamel mineral content. This soft enamel fractured from the occlusal surfaces of the primary molars (Hart et al., 2004). No other phenotype resulted from this nonsense mutation in *KLK4*. The second human *KLK4* mutation was recently discovered by use of whole exome sequencing which identified a single nucleotide deletion (p.Gly82Alafs^*^87) in both alleles of a 9 year-old female. The frameshift was in the third of five coding exons so the mutant *KLK4* transcripts may have been degraded by nonsense-mediated decay. If translated, the mutant protein would lack the same catalytic triad that was also lacking in the first discovered *KLK4* mutation. As for the previously discovered KLK4 mutation, the enamel covering this proband's teeth appeared normal in size and shape, but was discolored yellow-brown and chipped on multiple teeth. This proband was also secondarily affected with dental caries (Wang et al., 2013). No other phenotype was observed due to the nucleotide deletion in *KLK4*. Therefore, in humans KLK4 is essential for enamel to achieve its final hardened form, and that just as for MMP20, the only non-overlapping function of KLK4 is in dental enamel development.

## The *KLK4 knockout/LacZ* knockin mouse

**Figure 2:**
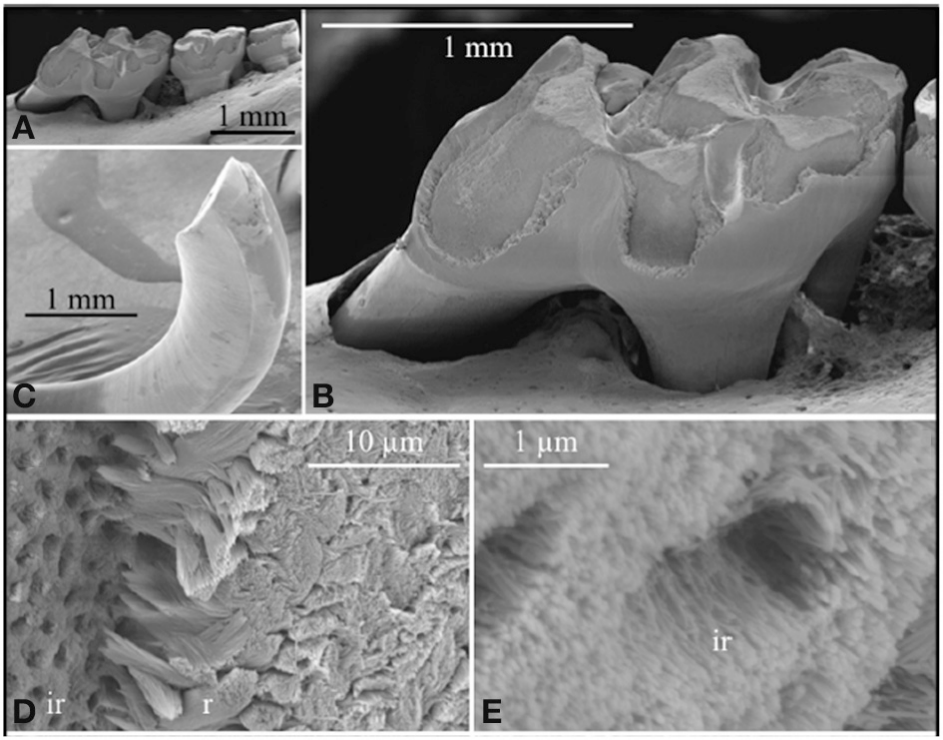
**Scanning electron micropscopy of the mandibular molars (A,B) and mandibular incisor (C–E) of a *Klk4* null mouse at 7 weeks**. The enamel of all molars showed a significant loss of enamel from all working surfaces (buccal cusps, occlusal surface, and marginal ridges) **(A,B)**. Similarly, the enamel layer was abraded at the working (buccal) surface of the mandibular incisor at its tip **(C)**. Higher magnification of the chipped area near the tip of the incisor showed the break was in the enamel layer, close to, but not at the DEJ. The broken surface appears to be composed of interrod (ir) enamel with holes where enamel rods (r) had pulled out and separated **(D)** from the initial deposit of interrod enamel near the DEJ. The holes are too numerous to be made by odontoblastic processes penetrating the enamel (enamel spindles). The orientation of the crystallites on the walls of the holes is parallel to the direction of the tubular holes and to the crystallites between the holes **(E)**. This figure was originally published by the American Society for Biochemistry and Molecular Biology in Simmer et al. (2009).

As stated above under KLK4 tissue localization, gene targeting was used to generate a mouse strain carrying a null allele of *Klk4* that has a nuclear *LacZ* reporter gene inserted directly into the *Klk4* translation initiation site. Therefore, the *LacZ* code was positioned in the same genomic context as wild-type *Klk4* and so provided a sensitive tissue reporter for native *Klk4* expression (Simmer et al., 2009). Other than a tooth phenotype, the *Klk4* ablated mice were normal. The teeth were normal, the enamel attained normal thickness and no abnormalities were observed until the enamel reached the transition to early maturation stage of development. At this point, the normal export of enamel matrix proteins from the matrix back to the ameloblasts destined for lysosomal degradation was impeded. The enamel retained proteins that were normally removed and the soft, protein-rich enamel abraded from the mouse teeth (Figures 2A–C). This strongly supports the belief that KLK4 functions to cleave enamel matrix proteins to facilitate their export out of the hardening enamel (Simmer et al., 2009). Unexpectedly, the rod enamel sometimes pulled away from interrod enamel. This left holes in the interrod enamel that were once filled by enamel rods (Figures 2D,E) (Simmer et al., 2009, 2011a). Backscatter scanning electron microscopy revealed that the enamel layer of *Klk4* null mice is reasonably well-mineralized at the surface, but is progressively less mineralized with depth (Hu et al., 2011; Smith et al., 2011). This pattern suggests that extracellular degradation of enamel proteins by KLK4 facilitates the movement of proteins in the deeper enamel toward the surface for ameloblast endocytosis.

**Figure 3:**
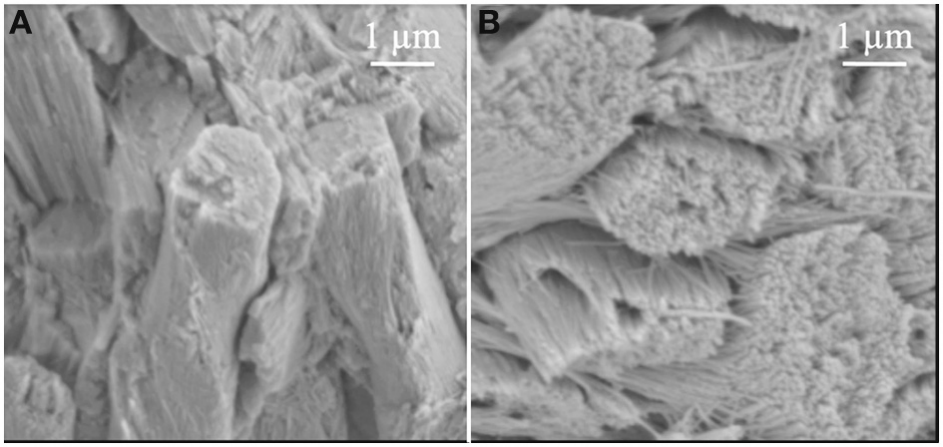
**Comparison of enamel rods from (A) wild-type and (B) *Klk4* null mice**. Enamel rods in wild-type mice have tightly packed crystallites that lose some aspect of their individuality. Enamel rods in the *Klk4* null mice are composed of distinctly individual crystallites resembling angel hair spaghetti. Holes or vacancies in some rods give the impression that smaller bundles of crystallites broke at a slightly deeper level and slid out of the rod. This figure was originally published by the American Society for Biochemistry and Molecular Biology in Simmer et al. (2009).

Another observation in the *Klk4* null mice was that the lack of KLK4 activity prevented the individual crystals within the rod from growing sufficiently in width and thickness so that they could interlock with adjacent crystals. Strikingly, bunches of crystals appeared to fall out of the rods and separate into individual crystals (Figure 3) similar in appearance to strands (crystals) of “uncooked angel hair spaghetti” falling out of a circular bundle (rod). Although the normal rod pattern was present in the *Klk4* ablated enamel, the ~10,000 crystallites within the rod failed to interlock properly and the crystallites fell from the rods (Simmer et al., 2009). Developmental analyses of *Klk4* null incisor enamel showed that percent mineral by weight increased almost identically to that occurring in wild-type mice until mid-maturation when a level of about 80% mineral by weight was attained. In contrast to wild-type enamel, the mineral content of the null mouse enamel remained unchanged as development progressed through the maturation stage (Smith et al., 2011). This seminal finding indicated that enamel maturation advanced normally, even in the presence of abundant protein, but arrested when the residual protein physically blocked final crystal maturation by occupying the shrinking space between crystals. If amelogenins and/or the other proteins inhibited crystal maturation by selectively binding to the sides of enamel crystals, the inhibition would have occurred earlier and would have resulted in a more severe enamel phenotype. All-in-all, the *Klk4* knockin/knockout mouse has revealed surprises about enamel formation and has forced us to reexamine some of our more firmly held beliefs about how crystallites grow in width and thickness and interlock to form an enamel rod.

## A modified theory of enamel development

During the secretory stage, proteins are secreted into the enamel matrix and are quickly cleaved by MMP20. Selected enamel protein cleavage products accumulate within the matrix and as new proteins are secreted they also are cleaved so that an abundance of MMP20 cleavage products are present throughout the enamel layer as the entire enamel layer grows away (thickens) from the dentin. Previously it was demonstrated that enamel mineral first forms as amorphous calcium phosphate (ACP) (Bodier-Houlle et al., 2000; Beniash et al., 2009). ACP has no defined structure. It can be thought of as “grains of sand” that require a mold if it is to have defined 3D structure. However, as described above, the enamel crystallites have a very specific shape. They grow into long thin ribbons. So it is postulated that the MMP20 cleavage products form a mold to define the shape of each crystallite ribbon so that the ACP can attain the proper shape prior to its conversion into HAP. Therefore, the nucleation event for mineral formation would occur within the protein mold so that the ACP will form a proper 3D ribbon structure prior to when it crystallizes into HAP. It is envisioned that the crystallite ribbon molds protect the crystallite ribbons, much as packaging material protects the contents of a box, as the enamel crystallites elongate from the dentin surface to the eventual outer surface of the enamel layer. This new theory (Simmer et al., 2012) represents a departure from previous beliefs that amelogenin by itself initiates enamel formation and from the thought that amelogenin inhibits crystallite growth in width and thickness (Bartlett and Simmer, 1999). In summary, results from the *Klk4* knockin/knockout mouse have led us to reevaluate our theories of enamel formation because we now have attained a better understanding of the importance of KLK4 activity and why it is so critical in enamel development.

### Conflict of interest statement

The Associate Editor declares that, despite being affiliated to the same institution as one of the authors, John D. Bartlett, the review process was handled objectively and no conflict of interest exists. The authors declare that the research was conducted in the absence of any commercial or financial relationships that could be construed as a potential conflict of interest.
